# Cures with a Single Dose

**Published:** 1891-11

**Authors:** J. R. Haynes

**Affiliations:** Indianapolis, Ind.; Clinical Bureau, I. H. A.


### CURES WITH A SINGLE DOSE.

J. R. Haynes, M. D., Indianapolis, Ind.

(Clinical Bureau, I. H. A.)

Belladonna.—Mrs. M., aged forty-two, dark complexion, black hair and eyes, rather small in stature, would weigh about ninety-five pounds, very nervous, perspires very easily, consequently takes cold at almost every change of the weather; has been subject to what she calls hay fever, and during the summer season has been in the habit of going to middle Michigan to spend the season. She reported that as soon as she would arrive there she would get instant relief, and would have no further trouble if she remained until after we had a heavy frost.

I was called to see her; found her in bed perspiring profusely; very restless; wanted to throw everything off in the way of covering; said that when in a perspiration she could not bear to be covered up and must throw the covers off. She complained of a sharp, cutting pain in the right side of the face, which would shoot up into the temple and side of the head, as if some sharp instrument was suddenly forced up through the side of the face; with a continuous, dead, heavy ache over the whole side of the face. When these sharp pains passed through the side of her face they would make her groan in spite of all of her efforts to keep quiet. The face was slightly flushed, tongue coated white with a red tip, and the papilla prominent, mouth dry, wanted water, but very little at a time, or just enough to wet the mouth; throat sore, looked red, with some difficulty in deglutition; sore pain under the sternum and extending over the whole front of the chest; gummed up sticky taste in the mouth; very gloomy, said that she must have immediate relief or she could not live, very gloomy and despondent. Pulse 130, small and thready; large drops of perspiration standing on her face; also all over her body.

One dose of Belladonna$^{10m}$, dry, on her tongue, with Sac-lac. in water, one teaspoonful every hour; which relieved the pain in twenty minutes, and she went to sleep and slept for four hours, and the next morning the trouble had all disappeared. She was left Sac-lac. and told to be very careful for a few days; there was no further trouble.

BELLADONNA.—Mrs. S., aged seventy-six, rather heavy set, light complexion, gray hair, blue eyes, generally enjoyed very good health for one of her age, was quite active and in good health, could do a good day's work about the house and not mind it, seemed to enjoy being busy.

Was taken suddenly with a severe chill, which was somewhat relieved by covering up in bed; from which she soon broke out with a drenching perspiration, which made her very restless; wanted to throw everything off, as the sweating made her feel so much worse.

Found her with a severe cough, hoarse and dry, would come on by paroxysms, which she could not control in the least, and if she attempted to talk it would bring on a paroxysm of coughing; tongue coated white, with a red tip and prominent papilla; mouth dry but no thirst; throat sore, bright red fauces, and when she coughed said that it felt as if a knife was being stuck into it; soreness under the sternum and across the front of the chest, aggravated by coughing or by touch, or attempting to take a full breath, which would bring on a spasmodic spell of coughing, which would last for several minutes.

Belladonna$^{10m}$, one dose dry, on her tongue, and Sac-lac. in water, one teaspoonful every hour; at the next call she was somewhat improved, had slept fairly well during the night; cough was looser, could talk without bringing on the paroxysms of coughing; but no desire for any kind of food; was not so restless, could bear to be covered in bed. Sac-lac. in water as before; there was a continuous improvement; the cough became loose, the sweating disappeared, appetite soon improved, and in five days she was pronounced well; but was cautioned that she must be careful for several days, and to let me know if she met with any mishaps. There was no further trouble.

HEPAR.—Mrs. S., aged thirty-one years, light complexion, light brown hair, blue eyes, rather tall and slim, would weigh about 105 pounds; rather nervous and fidgety; had taken a severe cold (la grippe) which she and some of her good neighbors had endeavored to cure, but without success; what had been given I could not find out; but any amount of trash, both internally and externally, had been used.

She had a deep, heavy, tight cough; what she raised was of a deep yellowish color, and said that it left a very putrid taste in her mouth; a dull, heavy headache over the whole head, worse in the forehead, aggravated by the cough; face was pale except when coughing, when she would flush up a pinkish color; tongue coated white; no particular bad taste in the mouth; breath putrid; very thirsty, wanted the water cold and in large quantities; no appetite for food, as nothing tasted natural; tenderness over the stomach and bowels, with a considerable flatulence; urine of sufficient quantity, but dark brown, with a strong smell; felt weak and prostrated, as if it was hard work to move even her arms or lower limbs, or to make any effort to stir; would much rather remain perfectly quiet; pulse 100 and rather soft; felt chilly upon every movement of her body, even in a warm room; soreness in the throat and through the whole chest, aggravated by coughing; all of her symptoms were ameliorated when in a perspiration; wanted the covering drawn up close around her neck to keep up the perspiration, as when it dried off it made her very restless and nervous.

Hepar$^{10m}$, one dose, dry, on her tongue, and Sac-lac., in water, one teaspoonful every hour.

At the next call there was some improvement, cough was easier, could raise the sputa with less trouble, less soreness in the chest, pulse 90, was not so particular about being covered up; had slept a part of the night, felt better on awaking; less thirst, tongue not so heavily coated, and not so nervous and despondent.

Sac-lac. in water, one teaspoonful every hour as before; close watch was kept over this case for five days, when she was able to be up and dressed. She got but one dose of Hepar$^{10m}$; was cautioned that she must be very careful for several days, and to let me know as soon as possible should anything new arise. There was no further trouble.

BARYTA-CARB.—Miss E., aged sixteen, dark complexion, black hair and eyes, rather chubby built; was employed as child's nurse.

Was brought to me to see if I could do anything for her. The family complained that she had such a fearful foot-sweat, which was such a nuisance that no one wished to have her in their house, and that she could not keep a place on that account.

It was extremely offensive, of a sickening, putrid character, so that it would soon scent the whole house. There were but a few symptoms that could be elicited from her or the family.

In her earlier days she had a large number of warts on her hand, which some old woman removed with some kind of an application, but what it was she did not know.

The whole plantar surface of the feet and up between the toes looked as if they had been soaked in warm water for a long time. The skin was white and the feet were tender, especially when she was on them. The odor was so offensive that I was glad to get rid of her as soon as it was possible.

The folks said that at times she seemed to be deficient in her memory; at others was bright as any one; would have spells of great despondency and grieve over the merest trifles; and would go off by herself and sob and cry as if she had no friends in the world.

She seemed to have but little confidence in herself; had a good appetite, slept well, complained of no aches or pains, and at times was cheerful as any one, but at others was very despondent, and they did not know what to do with her, as they wanted to treat her right.

She was given one dose of Baryta-carb.$^{10m}$, dry, on the tongue, and Sac-lac. for one week, to be taken in water. This was kept up for six weeks, when she was pronounced cured of all of her troubles.

The foot-sweat was completely removed, as well as her despondent spells. It has been some years and I have heard no further complaints in that direction.

